# Safety of the first dose of fingolimod for multiple sclerosis: results of an open-label clinical trial

**DOI:** 10.1186/1471-2377-14-65

**Published:** 2014-04-01

**Authors:** Alice Laroni, Davide Brogi, Vincenzo Brescia Morra, Leonello Guidi, Carlo Pozzilli, Giancarlo Comi, Alessandra Lugaresi, Renato Turrini, Debora Raimondi, Antonio Uccelli, Giovanni Luigi Mancardi

**Affiliations:** 1 Department of Neuroscience, Rehabilitation, Ophthalmology, Genetics, Maternal and Child Health, University of Genoa, Largo Daneo 3, 16132 Genoa, Italy; 2 Department of Neurological Sciences, University Federico II, Naples, Italy; 3 Neurology Unit, S. Giuseppe Hospital, Empoli, Italy; 4 Department of Neurology, University La Sapienza, Rome, Italy; 5 Department of Neurology, INSPE, Vita-Salute San Raffaele University, Scientific Institute San Raffaele, Milan, Italy; 6 Department of Neuroscience and Imaging, University G. D’Annunzio, Chieti, Italy; 7 Novartis Farma, Origgio, Varese, Italy

**Keywords:** Atrioventricular block, Bradycardia, Multiple sclerosis, Fingolimod, Safety, Tolerability

## Abstract

**Background:**

In patients with relapsing-remitting MS (RRMS) fingolimod prevents disease relapses and delays disability progression. First dose administration of fingolimod is associated with a transient, dose-dependent decrease in heart rate (HR) in the 6 hours after drug intake.

The aim of the study is to to assess safety and tolerability of the first dose of fingolimod in a cohort of Italian patients with RRMS without alternative therapeutic options.

**Methods:**

Open-label, single arm, multicentre study. After the first dose of fingolimod, patients were observed for 6 hours and had their vital signs monitored hourly. Extended on-site monitoring was provided when required.

**Results:**

Of the 906 patients enrolled in the study, most (95.2%) did not experience any adverse event (AE) following fingolimod administration. Cardiovascular AEs occurred in 18 patients and included bradycardia (1.3%), first-and second-degree atrioventricular block (0.1% and 0.2%), palpitations (0.1%), sinus arrhythmia (0.1%) and ventricular premature beats (0.1%). All events were self-limiting and did not require any intervention. Extended monitoring was required in 34 patients.

**Conclusions:**

These results, in a population who better resembled real-world clinical practice in terms of concomitant diseases and medications, are consistent with previous clinical trials and confirmed that the first dose administration of fingolimod is generally safe and well tolerated.

**Trial registration:**

EudraCT 2011-000770-60

## Background

Fingolimod (Gilenya™, Novartis) is an oral drug recently approved in Europe and the USA for the treatment of relapsing-remitting multiple sclerosis (RRMS). Phase II and III clinical trials have shown that fingolimod is superior to placebo and interferon beta-1a in preventing relapses; it also delays disability progression compared to placebo [1-3]. The mechanism of action of fingolimod is based on its functional antagonism of the receptors for sphingosine-1 phosphate (S1P) expressed by lymphocyte subpopulations. The binding of S1P to S1P receptors normally mediates the egress of lymphocytes from lymph nodes, so the binding and internalization of these receptors by fingolimod blocks this signalling and results in compartmentalization of circulating lymphocytes in the peripheral lymphoid organs. This strongly reduces the attack induced by the circulating myelin-reactive lymphocytes.

The interaction of fingolimod with S1P receptors in tissues outside the immune system can lead to side effects. More specifically, fingolimod is associated with a transient decrease in heart rate (HR) after the first dose intake, likely due to its interaction with S1P1 receptors expressed in atrial myocytes [4]. This transient decline in HR after the first dose of fingolimod was first observed in a clinical trial with renal transplant patients [5]. Phase II and phase III clinical trials in MS patients showed that treatment with fingolimod causes a decrease in HR within the 6 hours after the first administration (with a nadir at the fourth to fifth hour post dose), which is transient, dose-dependent and usually asymptomatic, and that first- and second-degree Mobitz type I atrioventricular block (AVB) may occur as a complication of the treatment [1-3]. A recent pooled analysis of data from three phase III clinical trials with fingolimod in MS showed that, in the 1212 patients treated with fingolimod 0.5 mg/day, symptomatic bradycardia (with dizziness and moderate somnolence) occurred in 0.6% of patients, and the incidence of AVB was 4.7% for first-degree AVB (versus 1.7% in the placebo group) and 0.2% for second-degree AVB Mobitz type I (versus 0 cases in the placebo group) [6]. In addition, two case reports have been published describing cardiac complications in patients with MS receiving fingolimod; one reporting transient asystole lasting for 7.5 seconds occurring 21 hours after the first dose in a subject who was under treatment with the antipsychotic drug risperidone [7], and another reporting endocarditis resulting in severe mitral regurgitation, atrial fibrillation, and congestive heart failure in a patient with MS, Leiden V mutation, psoriasis and sicca syndrome, where fingolimod was considered a possible contributor to the cardiac complications [8].

The cardiac safety profile of fingolimod was reviewed in 2012 by the US Food and Drug Administration (FDA) and the European Medicines Agency (EMA), prompted by a report in the post-marketing period of a sudden death, within the 24 hours following the first dose, of a patient who suffered from hypertension and was co-treated with the calcium-channel blocker amlodipine and the beta-blocker metoprolol [9,10]. The conclusion of this review was that the overall benefit/risk ratio of fingolimod is positive, although hourly check of HR and blood pressure (BP) and electrocardiographic (ECG) monitoring at the first dose (either continuous, according to the EMA, or pre-dose and 6 hour post dose, according to the FDA) is recommended. Also the co-administration of fingolimod and drugs which can cause bradycardia and QT prolongation should be avoided [9,10].

In the period when fingolimod had already been approved by the EMA but was not yet available in the local market in Italy, a clear ethical and clinical need appeared evident for all patients considered to be without alternative therapeutic options. Such patients were those with an unsatisfactory response or poor tolerability to interferon beta or glatiramer acetate and who developed an excessive risk of progressive multifocal leukoencephalopathy (PML) in association with natalizumab therapy (or those who refused to take any PML risk). In order to meet this important medical need, an open-label single arm study was initiated in Italy at the beginning of 2011 with the two fold objective of providing the above mentioned patients access to fingolimod and collecting safety and tolerability data in this population, which is broader than that of the pivotal trials in terms of concomitant diseases and medications received. Here we report the safety and tolerability results of the first dose of fingolimod.

## Methods

### Study design

The clinical trial CFTY720 DIT03 (EudraCT number 2011-000770-60) was a non-comparative, open-label, multicentre study of the administration of fingolimod in patients with RRMS for whom no alternative suitable therapy was available. All patients provided written informed consent. After a screening period of 14 days, all eligible patients started treatment with fingolimod at the baseline visit. Follow up visits to assess tolerability and safety were conducted at month 1, month 3, month 6 and then every 6 months, until the end of the study, which occurred when the market drug was available at the specific site. Expanded Disability Status Scale (EDSS) was also scored at baseline and at the end of the study, and any relapse which might have occurred was recorded. Only the safety and tolerability data associated with the initial dose of fingolimod are reported herein.

### Patients

Eligibility criteria included relapsing remitting disease course, age 18 years or older, no other therapy options, EDSS score of 0 to 6.5 (inclusive), minimum washout period of 3 months in case of previous treatment with natalizumab. Key exclusion criteria were history of chronic disease of the immune system other than MS, infections (active systemic bacterial, viral or fungal infections), history or presence of malignancy, uncontrolled diabetes or moderate to severe diabetic retinopathy, macular oedema, negative varicella-zoster IgG antibody status, severe cardiovascular disease, treatment with class Ia or class III antiarrhythmic drugs and uncontrolled arterial hypertension.

### First dose administration

After a screening period of about 14 days, during which patient eligibility was verified through blood tests, vital signs measurement (HR, systolic and diastolic BP), ECG recording and ophthalmic evaluation, the first dose of fingolimod was administered at the baseline visit. Vital signs were measured again prior to study drug intake. Patients were then observed for 6 hours after treatment initiation for signs and symptoms of bradycardia (first dose monitoring) and by checking HR and BP hourly. Extended on-site monitoring was required in cases where the patient had symptomatic bradycardia or the HR at 6 hours was the lowest value measured; monitoring continued until resolution of symptoms or increase in HR, according to the investigator’s clinical judgement. Patients with symptomatic bradycardia were required to take the second dose of fingolimod at the clinical centre.

Neither ECG pre and post dose, nor continuous ECG monitoring was mandated by the study protocol; ECG was performed at discharge or at any time during the 6 hours if clinically indicated and evaluated by a cardiologist. The study protocol did not include a definition for bradycardia, which was reported based on the judgment of the treating neurologist.

### Ethical requirements

This clinical study was performed in accordance with the ICH Harmonized Tripartite Guidelines for Good Clinical Practice, with applicable local regulations, and with the ethical principles laid down in the Declaration of Helsinki. The study was approved by the local Ethics Committees (Appendix 1). Written informed consent was obtained for the publication of individual clinical details.

### Statistical analysis

The population of the present analysis is all patients who received at least one dose of study drug. Since data were not normally distributed, the nonparametric Mann-Whitney U test was employed to detect inter-group differences. Fisher’s exact test was employed to compare the prevalence of concomitant treatments among groups. A p-value less than or equal to 0.05 was considered significant.

## Results

### Study population: baseline characteristics

In total, 906 patients were enrolled in this trial (CFTY720DIT03) and included in this analysis. Table 1 shows the baseline characteristics of patients included in this study. The mean age of the population was 39 years, and 36% were male. Patients had a mean duration of MS of 8.56 years and a mean EDSS score of 3.06. Approximately 25% of patients had previously received natalizumab.

**Table 1:** Baseline demographics and characteristics

**Variable**	**n** ***=*** **906**
Age, years	
Mean ± SD	39.10 ± 9.87
Median (range)	39 (18–64)
Gender, n (%)	
Female	579 (63.9)
Male	327 (36.1)
Age at MS diagnosis, years	
Mean ± SD	30.52 ± 9.52
Median (range)	29 (8–60)
Duration of MS, years^a^	
Mean ± SD	8.56 ± 5.97
Median (range)	7 (0–31)
Number of relapses in previous year	
Mean ± SD	1.36 ± 0.67
Median (range)	1 (1–5)
EDSS score	
Mean ± SD	3.06 ± 1.65
Median (range)	3.0 (0–6.5)
Treatment history, n (%)^b^	
Interferon beta-1a or -1b	445 (49.1)
Natalizumab	220 (24.3)
Glatiramer acetate	186 (20.5)
Azathioprine	19 (2.1)
Experimental drugs	7 (0.8)
Other	4 (0.4)
None	25 (2.8)

*EDSS* Expanded Disability Status Scale; *MS* multiple sclerosis; *SD* standard deviation.

^a^Time from MS diagnosis to enrolment.

^b^Last MS treatment.

Concomitant medical conditions included hypertension (34/906 patients; 3.8%), mood disorders (75/906 patients; 8.3%) and headache (31/906 patients; 3.4%). Fifteen per cent (136/906) of patients were treated at baseline with drugs which had the potential effect of decreasing HR or prolonging QT interval, as detailed later.

At the baseline assessment, data on BP and HR were available for 905 patients. Of these, 125 patients (13.8%) had a systolic BP ≥140 mmHg and/or diastolic BP ≥90 mmHg. Median HR before starting the treatment was 74 beats per minute (bpm) (±10.0) and 39 patients (4.3%) had a HR lower than 60 bpm.

### Adverse events during the first dose administration

The majority of patients (863/906; 95.3%) did not have any adverse event following the first administration. The remaining 43 patients (4.7%) had at least one adverse event during the first dose monitoring as reported in Table 2. Of the non-cardiac symptoms, headache was the most frequent (6/906; 0.7%), followed by nausea (5/905; 0.6%). Cardiovascular adverse events occurred in 18/906 patients (2.0%) and included bradycardia (12/906; 1.3%), first-degree AVB (1/906; 0.1%), second-degree AVB Mobitz type I (2/906; 0.2%), palpitations (1/906; 0.1%), sinus arrhythmia (1/906; 0.1%) and ventricular premature beats (1/906; 0.1%). Four out of 12 patients with bradycardia had symptoms related to HR decrease such as nausea, asthenia and vertigo. One serious adverse event occurred in one subject with second-degree AVB Mobitz type 1; although the patient was asymptomatic and the event spontaneously resolved, the patient was hospitalized and treatment was stopped. Fingolimod was discontinued in another patient with persistent bradycardia reported by the treating neurologist, although it was deemed mild (HR 59 bpm). Therefore, two out of 906 patients permanently discontinued the treatment due to an adverse event occurring during the first dose monitoring.

**Table 2:** Adverse events reported during first dose monitoring after fingolimod administration

**Adverse event, n (%)**	**n** ***=*** **906**
Cardiac disorders	18 (2.0)
Atrioventricular block first degree	1 (0.1)
Atrioventricular block second degree	2 (0.2)
Bradycardia	12 (1.3)
Palpitations	1 (0.1)
Ventricular premature beats	1 (0.1)
Sinus arrhythmia	1 (0.1)
Ear and labyrinth disorders	2 (0.2)
Vertigo	2 (0.2)
Gastrointestinal disorders	8 (0.9)
Diarrhoea	2 (0.2)
Nausea	5 (0.6)
Vomiting	1 (0.1)
General disorders and administration site conditions	7 (0.8)
Asthenia	4 (0.4)
Chest pain	1 (0.1)
Chills	1 (0.1)
Hyperpyrexia	1 (0.1)
Malaise	1 (0.1)
Infections and infestations	3 (0.3)
Influenza	1 (0.1)
Nasopharyngitis	1 (0.1)
Urinary tract infection	1 (0.1)
Musculoskeletal and connective tissue disorders	2 (0.2)
Pain in extremity	2 (0.2)
Nervous system disorders	11 (1.2)
Dysgeusia	1 (0.1)
Headache	6 (0.7)
Paraesthesia	1 (0.1)
Dizziness	1 (0.1)
Neuralgia	1 (0.1)
Somnolence	2 (0.2)
Eye disorders	1 (0.1)
Vision blurred	1 (0.1)

AVB details are provided in Table 3. The three cases of AVB were detected during the course of the 6 hour monitoring, since ECG was performed, as clinically indicated. In particular, in one case (Case 1) arrhythmic frequencies were detected at pulse assessment, while in another case (Case 2) the investigator observed a large decrease in HR (15 bpm). In the last case (Case 3) the patient underwent the ECG due to appearance of symptoms (asthenia) probably related to the decrease in HR. All three patients with AVB had received interferon treatment prior to fingolimod: patient 1 was treated with intramuscular interferon beta-1a for 6 months and started fingolimod after a 1-month washout period; patient 2 was treated with subcutaneous interferon beta-1a (22 μg) for 2 years, then with subcutaneous interferon beta-1a (44 μg) for an additional 2.5 years and started fingolimod after a 1-month washout period; patient 3 was treated with subcutaneous interferon beta-1a (22 μg) for 1 month, then with subcutaneous interferon beta-1a (44 μg) for an additional year and started fingolimod after a 3-month washout period.

**Table 3:** Details on the three cases of atrioventricular block (AVB) with onset after the first dose of fingolimod

**Gender, age (case number)**	**Event**	**CV medical history**	**Concomitant medications**	**CV baseline assessment**	**Onset of event, hours post dose**	**HR at onset (bpm)**	**Outcome**	**Action taken**
Female, 24	Second degree AVB, M1	None	Topiramate	Normal ECG	3	54 (arrhythmic)	Spontaneously resolved within 12 hours	Patient was hospitalized
(Case 1)				HR: 67 bpm				
				BP: 100/60 mmHg				
Male, 29	Second degree AVB, M1	Cardiac murmur	None	Abnormal ECG^a^	2	58	Spontaneously resolved within 12 hours	Patient was hospitalized. Study drug discontinued
(Case 2)				HR: 73 bpm				
				BP: 130/90 mmHg				
Female, 29	First degree AVB	None	Trazodone	Normal ECG	4	68	Spontaneously resolved the day after	None
(Case 3)				HR: 72 bpm,				
				BP: 112/75 mmHg				

AVB: atrioventricular block, BP: blood pressure, bpm: beats per minute, CV: cardiovascular, ECG: electrocardiogram, HR: heart rate, M1: Mobitz type 1.

^a^Deemed not clinically significant; slight delay in right AV activation.

### Extended monitoring

**Figure 1:**
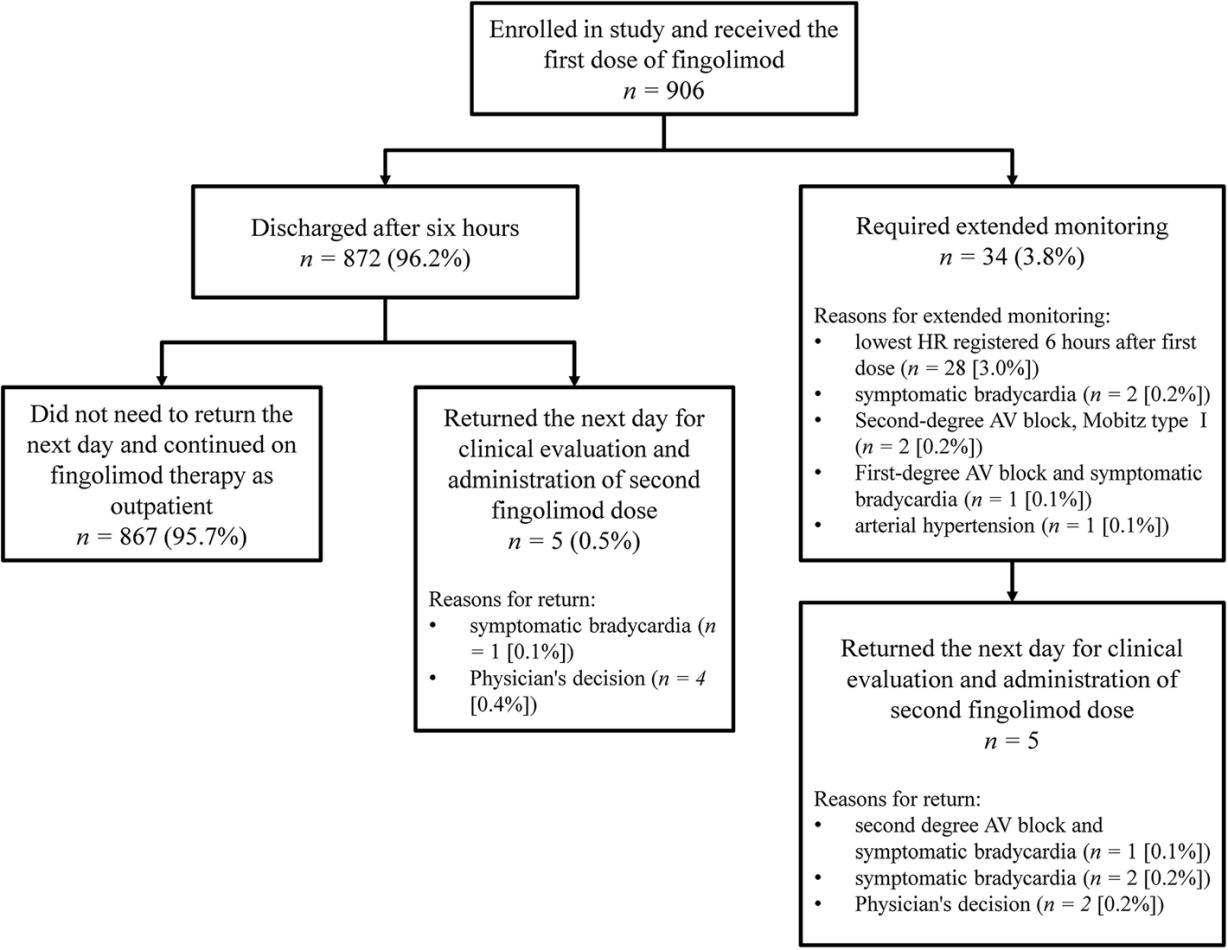
Patient disposition after first dose of fingolimod administration.

The majority of patients (867/906; 95.7%) was discharged after six hours and continued fingolimod therapy as outpatients. Extended monitoring was required in 34/906 (3.8%) patients due to: symptomatic bradycardia (2/906; 0.2%), lowest HR value at the sixth hour post-administration (28/906; 3.0%), first-degree AVB and symptomatic bradycardia (1/906; 0.1%), second-degree AVB Mobitz type I (2/906; 0.2%) or high BP (1/906; 0.1%). Five of these 34 patients also returned on the second day to repeat the procedure for the monitoring after dose intake. Baseline HR was not different in those who underwent extended monitoring versus those who were discharged at the sixth hour (mean values of 75.97 vs 73.97 bpm; p = 0. 436). Baseline systolic BP values were similar in the two groups (119.53 mmHg for those with extended monitoring vs 118.66 mmHg for those who were discharged at the sixth hour; p = 0.6380), as well baseline diastolic BP (77.59 mmHg vs 74.76 mmHg; p = 0.0704). Additionally, five subjects (0.5%) were discharged at the sixth hour without the need of extended monitoring but returned the next day for clinical evaluation and administration of the second dose (Figure 1).

### Concomitant medications

Table 4 describes the use of concomitant medications with a potential effect on cardiac function in the overall study population (906 patients) and in the population who underwent the extended monitoring (34 patients). No significant relationship between the concomitant treatment with beta-blockers, selective serotonin reuptake inhibitors (SSRI), tricyclic antidepressants, serotonin–norepinephrine reuptake inhibitors (SNRI) antidepressants, antipsychotic drugs, muscle relaxants, antimuscarinic drugs or macrolide antibiotics and the need for extended monitoring was found; while the use of topiramate, 4-aminopyridine and trazodone were found to be significantly associated with need for extended monitoring (p = 0.03, p = 0.01, p = 0.03 respectively), the patient numbers were small (2/34, 2/34 and 1/34, respectively). In detail, topiramate was taken by one patient who developed second-degree AVB and by one patient who was not discharged due to lowest HR at sixth hour (HR after 6 hours was 65 bpm), while two patients treated with 4-aminopyridine were not discharged due to lowest HR at sixth hour. Additionally, the only patient with first-degree AVB was the only subject treated with the antidepressant drug trazodone in the whole cohort. Given the small number of subjects, caution is warranted in interpreting these results.

**Table 4:** **Concomitant medications, n (%), in the total patient population (n** ***=*** **906) and in patients who underwent extended monitoring after the first dose of fingolimod (n** ***=*** **34)**

**Potential CV effect**	**Drug class**	**Drug**	**Total population**	**Patients who underwent extended monitoring**	P-value*
Bradycardia	Antihypertensive	Beta-blockers	6 (0.7)	0	NS
	Anti-epilepsy, anti-migraine	Topiramate	8 (0.9)	2 (0.6)	0.03
Prolong QT interval	Anti-fatigue	Amantadine	18 (2.0)	1 (0.3)	NS
		Modafinil	4 (0.4)	0	NS
		4-Aminopiridine	5 (0.5)	2 (0.6)	0.01
	Antidepressant	Tricyclics^a^	10 (1.1)	0	NS
		SSRIs^b^	58 (6.4)	1 (0.3)	NS
		Escitalopram	26 (2.9)	1 (0.3)	NS
		SNRIs^c^	35 (3.9)	2 (0.6)	NS
		Duloxetine	21 (2.3)	2 (0.6)	NS
		Trazodone	1 (0.1)	1 (0.3)	0.03
		Bupropion	2 (0.2)	0	NS
		Mirtazapine	3 (0.3)	0	NS
	Anti-psychotics		7 (0.8)	0	NS
	Muscle relaxants	Tizanidine	3 (0.3)	0	NS
	Antimuscarinic drugs	Tolterodine	2 (0.2)	0	NS
		Solifenacin	5 (0.5)	0	NS
	Antibiotics	Macrolides	1 (0.1)	0	NS
Other^d^	Benzodiazepines		39 (4.3)	2 (0.6)	NS
	ACE-inhibitors		14 (1.5)	1 (0.3)	NS
	Gabapentin		24 (2.6)	2 (0.6)	NS
	Levotiroxine		40 (4.4)	2 (0.6)	NS
	Tamsulosin		12 (1.3)	1 (0.3)	NS
	Baclofen		39 (4.3)	1 (0.3)	NS

ACE: angiotensin-converting enzyme, CV: cardiovascular, NS: not significant, SNRI: serotonin norepinephrine reuptake inhibitor, SSRI: selective serotonin reuptake inhibitor.

*P-value was calculated using the Fisher’s exact test.

^a^Clomipramine and amitryptiline.

^b^Citalopram, escitalopram, fluoxetine, paroxetine, sertraline.

^c^Duloxetine and venlafaxine.

^d^Includes all drugs taken by those who required prolonged monitoring excluded NSAIDs and acetaminophen.

## Discussion

We report safety and tolerability data on the first administration of fingolimod in a large cohort of Italian patients resembling “real world” patients. In such a patient population, the first administration of fingolimod was safe overall. Adverse events were reported for a minority of patients (4.7%) and were of mild severity. The only serious adverse event was one asymptomatic case of AVB second degree Mobitz type I that required hospitalization but resolved spontaneously. Only 2/906 patients discontinued the treatment at the first dose administration due to an adverse event (asymptomatic and spontaneously resolving AVB second degree, Mobitz type 1, and mild persistent bradycardia). A total of 18/906 patients reported cardiac adverse events. A small number of patients were not discharged after the first 6 hours, due to AVB, symptomatic bradycardia, or lowest HR registered at the sixth hour. It is important to underline that the episodes of AVB and symptomatic bradycardia were self-limiting and no patients developed life-threatening changes in HR. In all three cases of AVB, the patients had been previously treated with interferon; however, given the duration of the washout period in each case, and the short half-life of interferon (hours), we propose it is unlikely that the previous interferon treatment could have a synergistic role with fingolimod in causing AVB. Although it would have interesting to be able to analyse these data to further investigate the possible predisposing or synergistic role of a previous disease-modifying drug with fingolimod in causing AVB, the variability in previous treatment regimens, duration and washout periods, together with the limited number of AVB cases did not allow a reliable statistical analysis to be performed.

While randomized clinical trials performed during drug development are often sufficient to delineate the benefit/risk profile of a drug [11], the rate of rare adverse events may be underestimated due to the limited number of enrolled patients and to the particular selection criteria employed [12,13]. From this point of view, controlled clinical research has clear value in terms of intrinsic validity, whereas high extrinsic validity is associated with large trials or clinical experience in a real word setting. Patients in this study were older, had higher disease duration and lower mean relapse rate within the previous year in comparison to the populations treated with the same dose of fingolimod in phase III studies [1,3]. In these phase III studies, bradycardia was found in 0.9% and 0.5% of patients receiving fingolimod 0.5 mg/day, compared with 0.4% in the present study. First degree AVB was found in 0.5% and 0.2% of patients in the phase 3 studies, while 0% and 0.2% of patients demonstrated second degree AVB [1,3]. Table 5 compares the occurrence of extended monitoring and cardiovascular adverse events in this cohort to what was reported in a pooled analysis of patients treated with the same dose of fingolimod in phase III trials and from the US EPOC study [6,14]. This comparison shows again that these frequencies are consistent across the various studies, and confirms the very benign cardiovascular profile of fingolimod both in controlled clinical trials and in a real word experience like this study.

**Table 5:** **Clinical experience after one dose of fingolimod 0.5 mg/day in the present study (CFTY720DIT03) in comparison with a pooled analysis of three pivotal fingolimod phase III studies (2302, 2301, 2309 **[6]**) and the US EPOC study **[14]

**n (%)**	**CFTY720DIT03**	**2302, 2301, 2309 **[6]	**EPOC **[14]
	**n = 906**	**n = 1212**^**a**^	**n = 783**
Discharged at 6 hours, did not return for monitoring	872 (96.2)	1006 (83.0%)	772 (98.6%)
Required extended monitoring after 6 hours	34 (3.8)	157 (12.9%)	10 (1.3%)
	5 required 2 days of observation		2 required 2 days of observation
Required day 2 observation	10 (1.1)	32 (2.6)	3 (0.4)
Study drug discontinued	2 (0.2)	1 (0.1)	0 (0.0)
Symptomatic bradycardia	4 (0.4)	7 (0.6)	8 (1.0)
AVB, first degree	1^b^	56 (4.7)	15^b^
AVB, second degree	2^b^	2 (0.2)	0^b^

AVB: atrioventricular block.

^a^Only patients treated with fingolimod 0.5 mg/day included.

^b^The frequency of AVB was not calculated since ECG was not performed routinely in all patients and the exact number of patients who underwent to ECG was not captured.

About 1/3 of the large population included in this study had other medical conditions and a significant number of subjects (136/906, 15.0%) were treated with drugs that may influence HR and conduction. Importantly, the use of many medications which can cause bradycardia or influence the cardiac conduction, including beta-blockers or anti-psychotic drugs, was not associated to cardiac adverse events. Although we found some relationship between the use of topiramate, 4-aminopyridine and trazodone and the need for extended monitoring, the patient numbers were small and so firm conclusions cannot be drawn from this result. While it appears that the impact of existing cardiovascular-related medications on the adverse event profile of fingolimod is small, further investigation into the effect of these drugs on the safety and tolerability of fingolimod is warranted.

The strength of this study is the better generalizability of its results, obtained in a large population of patients resembling that of clinical practice in terms of concomitant diseases and medications compared the selected population of the pivotal phase III trials. Weaknesses include the lack of a standardized definition of bradycardia, and that ECG was performed only if clinically indicated and not routinely. While this different procedure may have led to missing some cardiovascular events, especially in asymptomatic patients, we believe that the likelihood of clinically relevant adverse events being missed is small, considering that patients were continuously followed for the first six hours through monitoring HR, blood pressure and clinical status. In addition, it should be considered that the clinical significance of cardiovascular events following pharmacological stimulation in patients with multiple sclerosis, who are otherwise generally healthy (such as those enrolled in this study), is different from that of patients with pre-existing cardiovascular disease. In this context, the clinical relevance of asymptomatic or mild symptomatic events, which may have been missed during the first dose monitoring, is likely very limited.

## Conclusion

Taken together, the results of this study, run in a population of patients with RRMS who did not have alternative suitable options and who better resembled real-world clinical practice in terms of concomitant diseases and medications, confirmed that the first dose administration of fingolimod is generally safe and well tolerated.

## Appendix 1

List of Local Ethical Committees:

Comitato Etico Dell’azienda Ospedaliera Universitaria San Martino di Genova, Genoa Italy; Comitato Etico per le Attività Biomediche Carlo Romano dell’Università degli Studi Federico II di Napoli, Naples, Italy; Comitato Etico dell’Università Sapienza, Rome, Italy; Comitato Etico per le Province di Chieti e Pescara, Chieti, Italy; Comitato Etico Area Vasta Centro, Azienda Ospedaliera Universitaria Careggi di Firenze, Florence, Italy; Comitato Etico dell’Ospedale San Raffaele, Milan, Italy; Comitato Etico Interaziendale A. O. Citta’ della Salute e della Scienza di Torino, Turin, Italy; Comitato Etico dell’Azienda Ospedaliera Policlinico Consorziale di Bari, Bari, Italy; Comitato Etico dell’Azienda Ospedaliero-Universitaria Ospedali Riuniti di Foggia, Foggia, Italy; Comitato Etico dell’Università Cattolica del S. Cuore-Policlinico Gemelli, Roma-Lazio, Rome, Italy; Comitato Etico dell’IRCSS Istituto Neurologico Mediterraneo–Neuromed di Pozzilli (Is), Isernia, Italy; Comitato Etico per la Sperimentazione Clinica delle Province di Verona e Rovigo presso Aqui Verona, Verona, Italy; Comitato Etico per la Sperimentazione Clinica della Provincia di Vicenza presso ULSS 6 Vicenza, Vicenza, Italy; Comitato Etico Aziendale dell’azienda Ospedaliera-Universitaria S. Maria della Misericordia di Udine, Udine, Italy; Comitato Etico Scientifico dell’azienda Ospedaliera Universitaria Policlinico G. Martino di Messina, Messina, Italy; Comitato Etico dell’Azienda Ospedaliera Universitaria S. Luigi Gonzaga di Orbassano, Turin, Italy; Comitato Etico Indipendente presso la Fondazione PTV–Policlinico Tor Vergata di Roma, Rome, Italy, Comitato Etico dell’Azienda USL 4 di Matera, Matera, Italy; Comitato Etico dell’Azienda Ospedaliera Ospedali Riuniti Villa Sofia-Cervello, Palermo, Italy; Comitato Etico dell’azienda Ospedaliera Universitaria Ospedali Riuniti Umberto I-GM Lancisi–G. Salesi di Ancona, Ancona, Italy; Comitato Etico della AUSL LE di Lecce, Lecce, Italy; Comitato Etico dell’azienda Ospedaliera S. Croce e Carle di Cuneo, Cuneo, Italy; Comitato Etico Lazio I presso Azienda Ospedaliera San Camillo–C. Forlanini di Roma, Rome, Italy; Comitato Etico dell’Azienda Ospedaliera Cardarelli Napoli, Naples, Italy; Comitato Etico per le Sperimentazioni Cliniche della Provincia di Padova presso AOU Padova, Padua, Italy; Comitato Etico dell’Azienda Ospedaliera-Ospedale di Circolo e Fondazione Macchi di Varese, Varese, Italy; Comitato Etico per la Sperimentazione Clinica delle province di Treviso e Belluno presso ULSS 9 Treviso, Treviso, Italy; Comitato Etico dell’azienda Sanitaria Unica Regionale delle Marche di Ancona, Ancona, Italy; Comitato Etico della Fondazione Istituto S. Raffaele Giuseppe Giglio di Cefalù, Palermo, Italy; Comitato Etico dell’Azienda Ospedaliera ’Ospedale Di Lecco’, Lecco, Italy; Comitato per la Sperimentazione Clinica dei Medicinali Area Vasta Nord Ovest presso Azienda Ospedaliero Universitaria Pisana di Pisa, Pisa, Italy; Comitato Etico Azienda Ospedaliera Spedali Civili di Brescia, Brescia, Italy; Comitato Etico Azienda Ospedaliero Universitaria della Seconda Università Degli Studi di Napoli, Naples, Italy; Comitato Etico dell’Azienda Ospedaliera di Rilievo Nazionale e di Alta Specializzazione Garibaldi di Catania, Catania, Italy; Comitato Etico dell’azienda Ospedaliera ’Ospedale S. Carlo’ di Potenza, Potenza, Italy; Comitato Etico della Fondazione IRCCS Istituto Neurologico Carlo Sesta di Milano, Milan, Italy; Comitato Etico Interaziendale della ASO S. Antonio e Biagio e Cesare Arrigo di Alessandria E delle ASL 19 di Asti, 20 di Alessandria, 21 di Casale Monferrato e 22 di Novi Ligure, Alessandria, Italy; Comitato Etico per Parma c/o Azienda Ospedaliero-Universitaria di Parma, Parma, Italy; Comitato Bioetico dell’azienda Ospedaliero · Universitaria Policlinico-Vittorio Emanuele di Catania per il POU Policlinico G. Rodolico dell’Università di Catania, Catania, Italy; Comitato Etico-Scientifico dell’azienda Ospedaliera Ospedale Niguarda Ca’ Granda di Milano, Milan, Italy; Comitato Etico dell’azienda Ospedaliera Universitaria Mater Domini di Catanzaro, Catanzaro, Italy; Comitato Etico di Area Vasta Romagna e Istituto Scientifico Romagnolo per lo Studio e la Cura del Tumori di Meldola (FC), Forlì-Cesena, Italy; Comitato di Bioetica della ASL di Sassari, Sassari, Italy; Comitato Etico dell’IRCCS Fondazione Don Carlo Gnocchi di Milano, Milan, Italy; Comitato Etico dell’Azienda Ospedaliera Universitaria Policlinico P. Giaccone dell ’Università degli Studi di Palermo, Palermo, Italy; Comitato Etico delle Aziende Sanitarie dell’Umbria, Perugia, Italy; Comitato Etico del Comprensorio Sanitario di Bolzano, Bolzano, Italy; Comitato Etico ASL Na/1 di Napoli; Naples, Italy; Comitato Etico della Provincia di Modena, Modena, Italy; Comitato Etico dell’Azienda Ospedaliera ’S. Giuseppe Moscati’ Di Avellino, Avellino, Italy; Comitato Etico del Comprensorio Sanitario di Merano, Bolzano, Italy; Comitato Etico della Provincia di Ferrara, Ferrara, Italy; Comitato Etico per le Province di L’Aquila e Teramo L’Aquila, Italy; Comitato Etico per la Sperimentazione Clinica del Medicinali Area Vasta Sud Est, Siena, Italy; Comitato Etico dell’Azienda Sanitaria Provinciale di Caltanissetta, Caltanissetta, Italy.

## Abbreviations

AVB: Atrioventricular block; EMA: European medicines agency; FDA: US Food and drug administration; PML: Progressive multifocal leukoencephalopathy; RRMS: Relapsing-remitting multiple sclerosis; S1P: Sphingosine-1 phosphate.

## Competing interests

A. La has received honoraria for lecturing or travel expenses for attending meetings from Biogen Idec, Novartis, Teva, and Merck Serono Pharmaceuticals. DB has received travel expenses for attending meetings from Biogen Idec, Novartis, Lundbeck, and Merck Serono Pharmaceuticals. VBM has received funding for travel, speaker honoraria, and research support from Sanofi-Aventis, Bayer Schering Pharma, Merck Serono, and Biogen Idec. LG has received research support from Almirall. CP has served on scientific advisory boards for and has received speaker honoraria from Novartis, Merck Serono, Biogen Idec, Bayer Schering Pharma, and Sanofi-Aventis. GC has received fees for consulting services from Novartis, Teva, Sanofi, Genzyme, Merck Serono, Biogen, Bayer, Actelion, Serono Symposia International Foundation, Almirall, Geneuro, Chugai and Receptos. He has also received fees for speaking activities from Novartis, Teva, Sanofi, Genzyme, Merck Serono, Biogen, Bayer, Serono Symposia International Foundation, Almirall, and Receptos. A Lu is a Bayer Schering, Biogen Idec, Merck Serono and Genzyme Advisory Board Member. She received travel grants and honoraria from Bayer Schering, Biogen Idec, Merck Serono, Novartis, Sanofi Aventis and Teva and research grants from Bayer Schering, Biogen Idec, Merck Serono, Novartis, Sanofi Aventis and Teva. Prof Lugaresi has also received travel and research grants from the Associazione Italiana Sclerosi Multipla and was a Consultant of “Fondazione Cesare Serono”. AU received financial support for research, honoraria for consultation, speaking or both at meeting for Genentech, Roche, Allergan, Merck-Serono, Sanofi-Aventis, Biogen Dompé, Biogen Idec, and Novartis. GM has received honoraria for lecturing, travel expenses for attending meetings, and financial support for research from Bayer Schering, Biogen Idec, Novartis, Teva, Sanofi-Aventis and Merck Serono Pharmaceuticals.

## Authors’ contributions

ALa, DB, VBM, LG, CP, GC, ALu, AU and GLM contributed to the generation of the data. ALa, DB, RT, DR, AU and GLM performed study data analysis and drafted the manuscript. All authors reviewed and approved the final manuscript.

## Pre-publication history

The pre-publication history for this paper can be accessed here:

http://www.biomedcentral.com/1471-2377/14/65/prepub
